# A giant orbital solitary fibrous tumor treated by surgical excision: a case report and literature review

**DOI:** 10.1186/s13000-023-01350-8

**Published:** 2023-05-05

**Authors:** Qi Zhou, Yuting Liu, Fang Wang, Yang Cao, Hongbin Lv, Xibo Zhang

**Affiliations:** Department of Ophthalmology, Affiliated Hospital of Southwest Medical University, 25 Taiping Street, Sichuan 646000 Luzhou, China

**Keywords:** Solitary fibrous tumor, Orbit, Spindle cell tumors, Benign, CD34

## Abstract

**Background:**

Spindle cell tumors, called solitary fibrous tumors (SFTs), are of mesenchymal origin, and can develop in the orbit. As ‘intermediate malignancy’ tumors, only a small percentage show malignant behavior, such as invasion of surrounding tissue.

**Case presentation:**

A 57-year-old woman presented with a 19-year history of a giant right orbital mass. Orbital computed tomography (CT) revealed an inhomogeneously-enhancing mass compressing and engulfing the eyeball and optic nerve. She underwent lid-sparing orbital exenteration. Microscopic characteristics and immunohistochemistry (IHC) tests were indicative of a benign SFT. No recurrence was observed at the 4-year follow-up.

**Conclusion:**

Early and complete tumor resection is recommended.

## Background

Solitary fibrous tumors (SFTs), rare spindle cell tumors, were first described by Klemperer and Rabin in 1931 [1]. They usually occur in the pleura, pericardium, respiratory tract, peritoneum or mesentery, orbit, breasts, other soft tissues, and visceral organs [2]. Since Westra et al. [3] reported the first orbital SFT in 1994, increasing orbital involvement has been reported. Although most cases typically present as a slow-growing orbital mass and behave in a benign fashion, a few exhibit malignant behavior, such as recurrence and local invasion [2]. This report details a case of a giant orbit SFT compressing and engulfing the eyeball. To the best of our knowledge, this is the largest benign orbital SFT ever reported.

## Case presentation

In June 2018, a 57-year-old woman presented with a massive, painless mass in the right orbit which had developed over 19 years. As the tumor grew, vision in the right eye gradually deteriorated until it was completely lost approximately 15 years before presentation. Due to budgetary constraints, the patient avoided seeking appropriate medical care.

**Fig. 1:**
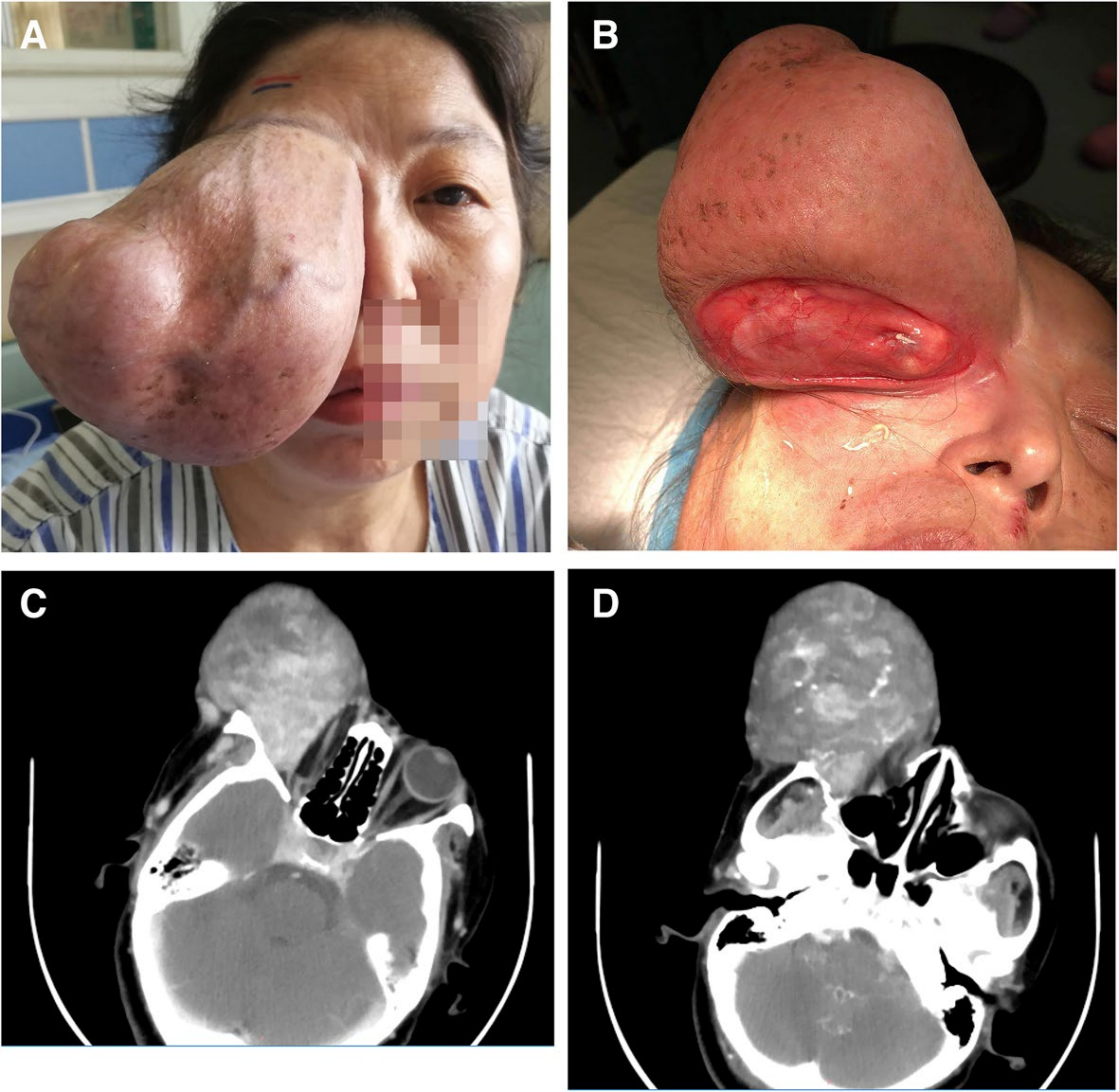
Preoperative photograph and findings of imaging studies. **A** A large multi-lobulated mass in the right orbit. **B** The palpebral fissure had widened, and the tumor had displaced the eyeball anteromedially (arrow). **C** Axial CT of the orbit shows a heterogeneous orbital mass. **D** Contrast-enhanced CT shows inhomogeneous and marked contrast enhancement of the mass. CT; computed tomography

Upon ophthalmological examination, she had a best-corrected visual acuity of no light perception and 20/20 of the right and left eyes, respectively. In the right orbit, a large rubbery mass stretched horizontally from the bridge of the nose to the lateral canthus, and vertically from the eyebrow to the center of the cheek. The upper eyelid covering the tumor was pushed forward due to the large size of the mass. The vessels of the eyelids were dilated and tortuous (Fig. 1A). The palpebral fissure was elongated and widened; the eyeball, covered with soft tissue, was displaced anteromedially (Fig. 1B). No abnormalities were observed in the left eye. There was no associated lymphadenopathy and a systemic examination revealed no abnormalities.

Orbital CT revealed a huge mass in the right orbit measuring 10.6 × 9.5 × 11 cm. After administration of contrast material, inhomogeneous enhancement and vascular-like enhancing structures were observed. The mass was closely related to the internal rectus muscle. The eyeball appeared totally engulfed and compressed by the large mass and was displaced anteromedially. In the bone structures, no evident erosion or extension damage was found; however, the temporal orbital wall was observed to be slightly thinner than anticipated (Fig. 1C-1D).

Orbital cavernous hemangioma was suspected. The patient underwent lid-sparing orbital exenteration under general anesthesia, a procedure that involved removal of the tumor, globe, orbital contents, and most of the upper eyelid. Using a preserved part of the normal eyelid skin, the orbital cavity was covered and sutured with a skin incision margin.

**Fig. 2:**
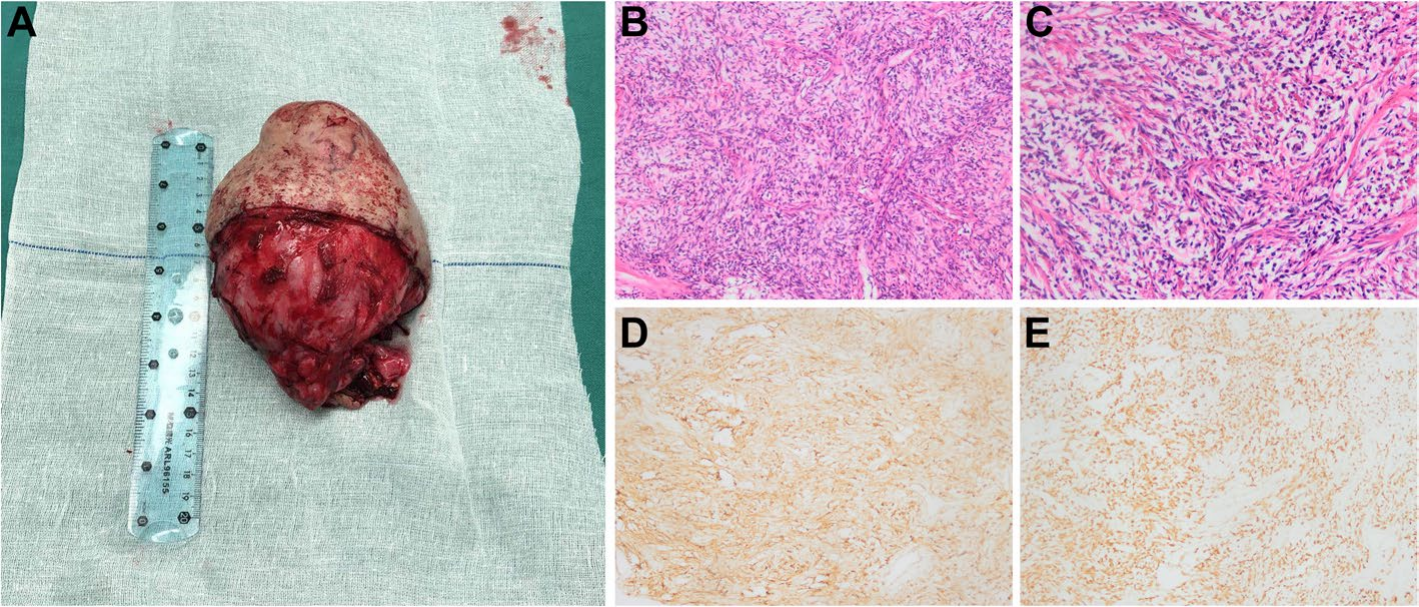
**A** Photograph of the excised lesion. 15 × 13 × 9 cm tumor covered with a 16 × 14 cm flap. **B** Densely arranged spindle-shaped cells. H&E stain, original magnification × 100. **C** Densely arranged spindle-shaped cells. H&E stain, original magnification × 200. **D** CD34, original magnification × 100. **E** STAT6, original magnification × 100. STAT6: signal transducer and activator of transcription 6

On gross pathological examination, the orbital lesion consisted of a 15 × 13 × 9 cm well-circumscribed solid tumor covered with a 16 × 14 cm flap (Fig. 2A). The cut surface was composed of gray-white or gray-red soft tissue with foci of hemorrhage identified in some areas. Nerve tissue of approximately 2.5 cm in length and 0.2 cm in diameter was observed in the adipose tissue next to the tumor. On microscopic examination, H&E staining showed that the tumor tissue constituted spindle-shaped cells arranged in bundles or irregular shapes. Cell nuclei were oval or spindle-shaped. No necrosis, mitoses, or nuclear pleomorphisms were present. (Fig. 2B–2C).

IHC testing demonstrated strong and diffuse spindle cells with antibodies against CD34, signal transducer and activator of transcription 6 (STAT6) (Fig. 2D-2E), B-cell lymphoma 2 (BCL-2), and progesterone receptors (PR). Approximately 2% of these cells were reactive to Ki-67. Staining for antibodies against P53, glial fibrillary acidic protein, and S-100 was negative. These findings were compatible with a diagnosis of a benign SFT.

The patient had good cosmetic results and exhibited no symptoms of recurrence during the 4-year follow-up.

## Discussion and conclusions

Patients with orbital SFTs are predominantly middle-aged adults [4, 5]. There was no apparent sex predilection for SFT. Any orbital space, including the intraconal and extraconal spaces of the orbit, can be affected by orbital SFTs. Lesions occurring in other tissues, such as the conjunctivae, lacrimal gland fossa, lacrimal sacs, eyelid, and pigmented outer layer of the pars plana of the ciliary body, have also been reported [6–10].

Symptoms and signs are related to tumor size and location. Patients with orbital SFTs often present with proptosis, eyelid swelling, blepharoptosis, diplopia, ocular motility restriction, and a slow-growing painless palpable mass in the periocular area [11]. Headaches and epiphora are rare symptoms. Visual acuity is generally normal or mildly impaired; however, if the optic nerve is involved, patients may develop significant reduction in visual acuity and even blindness of the affected eye. Fundus examination is usually unremarkable, but some patients show dilated vessels, optic disc and macular edema, and optic nerve atrophy due to elevated intraorbital and intraocular pressure [12, 13]. In our case, the patient presented with a painless mass for more than 10 years without medical intervention, which resulted in significant disfiguration and blindness of the right eye.

The reported radiological features of SFT are non-specific. Orbital SFTs are seen as well-circumscribed soft tissue tumors with moderate to intense enhancement on CT images, which are attributed to the high vascularity within the tumor [14]. Although extremely rare, bony erosion should prompt suspicion of a malignant tumor. On MRI, tumors have been demonstrated to have T1-weighted signal isointensity and T2-weighted isointensity to hypointensity, reflecting differences in the amounts of cellular components, collagen, and fibroblasts among different tumors [15]. They may be difficult to distinguish from tumors with high blood flow, such as fibrous histiocytomas, neurofibromas, hemangiomas, and schwannomas on CT and MRI. However, these imaging modalities may help with localization, tumor sizing, planning of surgical intervention, and postoperative monitoring. Complete en bloc excision is required to reduce the risk of recurrence [16]. In this case, orbital CT revealed that the giant tumor occupied virtually the entire orbit and extended beyond the orbit, entirely disrupting the normal structure and function of the eyeball and the optic nerve, ultimately leading to blindness. Therefore, an orbital exenteration was required to thoroughly remove the tumor.

Benigh SFTs typically exhibit low mitotic activity and lack nuclear pleomorphism and/or necrosis [17]. Histomorphological features of malignancy include increased mitotic activity (≥ 4/10 HPFs or > 2 mitoses/2 mm2), nuclear pleomorphism, tumor necrosis, increased tumor size (≥ 5 cm), and infiltrative borders [17, 18].

Microscopic features alone are insufficient to confirm the diagnosis of SFT and further IHC analysis must be conducted. SFT cells typically stain positive for specific markers, such as CD34 and STAT6, and variably positive for vimentin, S-100 protein, progesterone receptors (PR), P53, BCL-2, and Ki-67 [19]. CD34, an antigen expressed on endothelial cells and hematopoietic progenitor cells, stained strongly and diffusely, and it is believed to be the most diagnostic immunohistochemical biomarker for benign SFTs. CD34 negative or weakly expressed cells may be associated with malignant transformation [13]. Nuclear STAT6 overexpression is a highly sensitive and specific biomarker for SFTs; thus, SFTs can be distinguished from other orbital fibroblastic tumors [20]. Steroid hormone receptors and PRs are expressed in SFTs. Previous studies have reported both increased and decreased expression of PR in predicting high-risk behavior. Bongiovanni et al. [21] reported that PR positivity is a feature of pleura SFTs, demonstrating increased proliferative activity and a propensity for recurrence, while Carretta et al. [22] reported that lower expression of PR identifies pleura SFT with a higher risk of recurrence after surgery. Additional studies are still needed to confirm the effect of PR expression in orbital SFTs on predicting prognosis. In adult mammalian tissues, BCL-2 protein has a restricted pattern of expression which is limited to proliferating cells, stem cells, and hormone-responsive tissues. The presence of BCL-2 in this case may be related to the expression of PRs in neoplastic tissue [8]. According to Sun et al., Ki-67, a protein linked to ribosomal RNA synthesis and cell proliferation, can be used as a prognostic marker of SFTs and is diagnostically relevant for the assessment of malignant SFTs [23]. In benign SFTs, the Ki-67 index frequently reacts positively in 0–2% of spindle-cell nuclei; this proportion can increase to 40% in malignant tumors. A tumor suppressor gene, p53, plays a critical role in regulating cell proliferation. p53 is strongly expressed in SFTs with fatal outcomes, such as clinical recurrence, local invasion, and metastasis [24, 25]. In this case, CD34 and STAT6 immunoreactivity supported the diagnosis of SFT. Low expression of both p53 and Ki-67 was consistent with the tumor's histological features. Therefore, the features of microscopic features and IHC analysis in this case support the diagnosis of benign SFT.

The clinical behavior of SFTs is variable [16]. The majority of SFTs are slow-growing masses that pursue a nonaggressive course; however, clinical and radiological features are not necessarily associated with histological signs of malignancy [9]. Even in cases of benign tumors, aggressive clinical behaviors, such as adjacent tissue invasion [26–28], recurrence [11, 29, 30], metastasis [31], and malignant transformation [2, 4], have been demonstrated. Malignant SFT may occur de novo or by transformation within benign or low-grade tumors [24]. Local recurrence is usually attributed to incomplete initial resection of the tumor, which then shows a tendency to spread into the orbital bone or extra-orbital soft tissue [27, 32]. The giant and slow-growing orbital SFT in our case showed an indolent course of growth and did not display any aggressive behavior during the follow-up period. However, Demicco EG et al. [33] reported a risk prediction model for SFTs incorporating patient age, tumor size, and mitotic activity to predict risk of metastasis. Low-risk patients did not acquire any metastases, whereas the intermediate-risk group had a 7% 10-year metastatic risk and the high-risk group had a 49% 5-year metastatic risk. According to the criteria above, this case can be regarded as at intermediate risk. Regular long-term follow-up is necessary, even though our case did not show any evidence of tumor recurrence after a 4-year follow-up period.

Complete resection is necessary for adequate local tumor control. Other adjuvant treatments, including preoperative transarterial embolization, radiotherapy, and chemotherapy for the treatment of recurrent orbital SFTs, have been reported in individual cases [2, 34, 35]. The potential benefits of adjunctive therapy should be further evaluated in clinical trials.

In conclusion, we present an extremely rare case of orbital SFT which was definitively treated with surgical excision. To the best of our knowledge, this is the largest orbital SFT reported thus far. The tumor exhibited an aggressive course of eyeball compression and had typical histomorphological and IHC features indicative of a benign SFT. Therefore, careful, long-term follow-up is necessary.

## Data Availability

Not applicable.

## Abbreviations

SFT: Solitary fibrous tumor
STAT6: signal transducer and activator of transcription 6
BCL-2: B-cell lymphoma 2
PR: progesterone receptors
